# Progress in Surgery

**Published:** 1900-04-28

**Authors:** 


### PROGRESS IN SURGERY.

### GENERAL SURGERY.

(*Continued from page* 52.)

**Fractures and Dislocations (Upper Extremity).**—E. Eliot, jun.,[15] relates an interesting case of operation for deformity following supra-condyloid fracture of the humerus, which it was found impossible to reduce. The accident occurred in a boy, and there was the usual displacement backwards of the condyles of the humerus with the elbow joint, while the upper fragment was displaced anteriorly. Even after incision on the posterior aspect of the joint through the triceps muscle, reduction was impossible because of the overriding of the fragments. An anterior incision was therefore made through the skin, and the lower end of the upper fragment found beneath it, having penetrated the brachialis anticus. The expanded end of the humerus was thus button-holed. After this had been trimmed with a ronguer, reduction was effected. The wound suppurated apparently from the catgut sutures, but this subsided in 10 days. Three months later complete union had taken place. Five months after the original injury, and three months after the removal of the splint, pronation and supination were practically perfect. Flexion was slightly limited, but extension was normal. Mouchet[16] gives a useful account of fractures of the elbow, particularly in children. In 122 cases submitted to a radiographic examination, the author found the following fractures: Lower end of humerus, 103 cases; olecranon, 6; neck of radius, 5; coronoid process, 1. In the remaining seven cases the elbow was dislocated backwards. One fact especially noticed was that the fractures were only rarely true epiphysial separations, particularly in the bones of the forearm. Fracture of the neck of the radius was met with almost as frequently as fracture of the olecranon. The comparative infrequency of the latter in children, as compared with adults, is attributed to the small size of the process in children, and to the want of full development of the muscular system. The author's experience agrees with that of other observers that true epiphysial separation of the lower extremity (incompletely ossified) of the humerus is very rare after the age of four years. Carl Beck's[17] experience of 62 skiagraphs of fracture of the lower end of the radius bears out that of others, that simultaneous fracture of the styloid process of the ulna is present in the greater majority of cases. He thinks that a real chondro-epiphysial separation can take place in infants, though extremely rare. He classifies the different fractures of the lower end of the radius as: Epiphysial separations, fissures (infractions), complete and incomplete fractures, fractures of the lower end of the radius combined with infraction or fracture of the head of the ulna, and fracture of the lower end of the radius combined with fracture of the styloid process of the ulna. All these varieties may be extra-articular as well as intra-articular. Destot and Gallois[18] have experimentally studied these fractures, and depicted them with the X-rays. They divide them into three groups: (1) The epiphysis is detached and displaced in various positions. There is practically no intra-articular fracture. (2) The fragment is a bevelling of the posterior surface, while the anterior is left intact —described by Rhea Barton. (3) The epiphysis is crushed by the penetration of the diaphysis, and the fragments are separated from one another. These three forms are capable of numerous variations. After reduction under an anæsthetic of the displaced fragments in Colles's fracture, A. E. Rockey[19] maintains approximation by means of short lateral splints made of strips of pasteboard on the ulnar and radial sides of the wrist. A gap is between them front and back to protect the flexor and extensor tendons from pressure, and prevent interference with the circulation. He has used this method in 25 cases with great satisfaction. J. Elliot[20] records an interesting case of dislocation of the carpus with the hand backwards in a feeble woman, aged 64, from a fall on the palm. The carpal bones formed a prominence over the posterior surfaces of the radius and ulna—the lower ends of which were very prominent in front, while the palm of the hand was shortened. Reduction by extension was easy, but the bones slipped out again. After a second reduction ordinary anterior and posterior splints were applied with a pad over the dorsum of the wrist. A. J. Rodocanachi[21] mentions an unusual injury to the wrist of a cyclist. A diagnosis was made that the styloid process of the ulna with the triangular fibro-cartilage and carpus had been dislocated forwards, consequent on a fracture at the root of the styloid process, whereas the ligaments on the radial side had held. The deformity caused by the head of the ulna on the dorsal aspect could not be readily reduced, and the patient declined the employment of an anæsthetic and further treatment. A case of luxation of the semilunar bone, similar to another previously recorded by the author, is recorded by Berger.[22] In both there was swelling over the inferior radio-ulnar joint and beneath the flexor tendons, a clenched position of the hand, and the fingers could not be fully extended or flexed. There was also loss of sensibility in the distribution of the ulnar nerve. The diagnosis was confirmed by the X-rays. Extirpation of the displaced bone is the only treatment. Alfred King[23] gives skiagraphs and a description of a rare instance of anterior dislocation of the carpal scaphoid before and after operation. This was performed by open incision and replacement in its normal position two months after the accident, and after several unsuccessful attempts at reduction. The radial articular surface was found to be directed forwards, having been rotated in dislocating.

**Lower Extremity.**—Percy R. Bolton[24] describes the case of a boy who had sustained a fracture of the ilium and neck of the femur after a fall from a fourth story window. It was put up in a plaster spica, but at the end of five weeks it seemed as though displacement of the fragments of the femur was present. An anterior incision was made into the hip joint, and a complete fracture of the neck found, one and a-half inches from the margin of the cartilage. The periosteum was torn through the whole circumference of the neck. As there was no union, the head of the bone was excised with a satisfactory result. The boy could now get about with great freedom, and three-quarter inch shortening and flexion 90 degrees. A perfect recovery ensued in a case of multiple compound fracture, viz., compound fracture of the lower third of femur with protrusion of shaft, compound fracture of lower jaw, and compound fracture of the frontal bone, is recorded by H. A. Lediard[25] in a man aged 21. Any one of these fractures was serious in itself. After careful cleansing and antiseptic treatment of the wounds, the thigh was put up with extension and an interrupted long splint, with the ultimate result that at the end of five months there was only one-half inch shortening, and the patient reported that he was not incapacitated in any way. As an appendix to their paper in the Transactions of the Royal Medical and Chirurgical Society, H. L. Barnard and J. Hutchinson, jun.,[26] add four cases of separation of the lower epiphysis of the femur (two of them compound) treated by the method of reduction by full flexion of the knee. In 1884 Mr. McGill, of Leeds, employed this method of reduction by forcible flexion of the limb till the heel touched the buttock, and the present authors have further elaborated and extended this principle of full flexion by keeping the knee in this position for 10 days, often without the use of a splint. The first case quoted by them was a compound one from the London Hospital, where the limb was afterwards, on the second day, amputated by F. S. Eve for commencing gangrene due to ruptured popliteal artery and vein. Two skiagrams of the second case, before and after reduction of the epiphysis by the method just stated, show its efficiency. A skiagram of the third case showed union with the usual deformity due to displacement forwards of the epiphysis. The condition was recognised too late for replacement. The fourth case, one of compound separation, under the care of F. S. Eve, was reduced by direct pressure. The skiagram made whilst the limb was fully extended shows some gaping between the epiphysis and diaphysis behind. A very good recovery ensued. Vallas[27] thinks that a bad result from transverse fracture of the patella is due to laceration of the anterior and lateral portions of the capsule of the knee joint rather than to non-union with wide separation of the fragments. Fractures involving the bone alone should be treated as an uncomplicated hæmarthrosis of the knee, by compression, massage, and early movement of the limb. But patellar fractures with laceration of the fibrous capsule necessitate operation, which consists in free exposure of the seat of injury by a crucial incision, removal of blood from the joint by irrigation, and careful closure, by suturing, of the rents in the fibrous investment in front of the knee. In this way suturing of the bony fragments is held to be useless and unnecessary. A. A. Hudson[28] records a very successful case of simple fracture of the patella treated by wiring, in which between two and three months after the operation the patient—a young man of 25—could ride and walk all day, and even buck-hunt in South Africa. The writer of an article in the New York *Medical Record* recommends[29] the use of catgut or other absorbable sutures in bringing the fragments together. In his opinion, silver wire and thick silk are not required. Great tension is not needed to hold the fragments in position. As the result of his experiences, W. J. Means[30] concludes that the results of non-operative treatment of fractured patella are unsatisfactory, both as to long confinement and functional disability, while the methods of maintaining apposition of the fragments by external appliances are unsatisfactory and unscientific. In open arthrotomy the fragments can be carefully approximated and sutured so as to maintain apposition, and ultimately bony union. It saves months of confinement, and gives permanent results. The suture material should be absorbable, such as catgut or kangaroo tendon. The field of operation should be continuously irrigated with a hot salt solution during the manipulation, and the incision closed without drainage. The massage treatment begun at an early date is an important factor in restoring the functional activity of the joint. A rare instance of complete antero-posterior dislocation of both knees is described by H. E. Haycock[31] in a man aged 31 years, due to being caught in the revolving wheel of some machinery. Reduction was readily effected, and both joints resumed their natural shape. Back splints were applied. Four months after the injury the patient had complete power of flexion and no pain in the joints.

[15] Annals of Surgery, July, 1899, p. 93. [16] Med. Chron., Aug., 1899, p. 358; and Gazette des Hôpitaux. [17] New York Med. Jour., Sept. 9, 1899, p. 362. [18] Amer. Jour. of Med. Sci., May, 1899; and Rev. de Chirurg. [19] New York Med. Rec., July 29, 1899, p. 159. [20] Lancet, May 27, 1899, p. 1,431. [21] Lancet, Nov. 4, 1899, p. 1,229. [22] Epitome of Brit. Med. Jour., Aug. 15, 1899; and Bull. et Mem. de la Soc. de Chir., 1899. [23] Annals of Surg., Aug., 1899, p. 213. [24] Annals of Surg., May, 1899, p. 622. [25] Lancet, Nov. 25, 1899, p. 1,440. [26] Lancet, May 13, 1899, p. 1,275; [27] Epitome, Brit. Med. Jour., Jan. 20, 1900, p. 10; and Revue de Chirurg., 1899. [28] South African Med. Jour., May, 1899, p. 15. [29] New York Med. Rec., June 24, 1899, p. 911. [30] Columbus Med. Jour., July, 1899, p. 1. [31] Lancet, Aug. 5, 1899, p. 342.

